# Population finiteness is not a concern for null hypothesis significance testing when studying human behavior. A reply to Pollet (2013)

**DOI:** 10.3389/fnins.2015.00081

**Published:** 2015-03-12

**Authors:** Tadeg Quillien

**Affiliations:** Laboratoire Dynamique du Langage, Evolution, Cognition and Culture, Universite Lyon 2Lyon, France

**Keywords:** inferential statistics, *p*-value, methodology, macrolevel data, empirical research

Pollet (2013) recently questioned the use of Null Hypothesis Significance Testing (NHST) in cross-cultural research, when researchers sample most or the totality of the population of macro-level units they study. According to his interpretation, if a researcher is interested in, say, the correlation between two variables at the level of US States and includes data from all 50 US states in his sample, then he cannot coherently provide a *p*-value, since the latter only makes sense for samples that are proper subsets of the whole population.

This claim rests on the implicit assumption that a researcher who is interested in for example the 50 US states is working on a finite population, i.e., that she will have sampled the whole population of interest if she includes data from all 50 states in her analysis. While this assumption is a deeply intuitive one, it is also false in most cases.

In many textbook examples of statistics and probability, it does make sense to assume that we are working with a finite population. If an urn contains 100 colored balls, and the aim of the exercise is to make inferences about the proportion of red and blue balls within the urn on the basis of random samples, then it is safe to consider that the 100 colored balls within the urn constitute the whole population of observations. Thus, if we were to draw 100 balls from the urn and count more blue than red balls, it would not make sense to conduct NHST to check whether there are *significantly* more blue than red balls.

In other cases, however, we cannot assume that the statistical population we study is finite (see Howell, 2010, p.2). For instance, if we want to determine whether a die is fair based on a series of die rolls, we reason as if the population was infinite. The series of die rolls we are studying is considered a sample from the set of all possible rolls made with this die, and this set is intuitively infinite (or at least very large). Pollet's argument rests on the assumption that the discussed empirical research is fundamentally different, when it comes to statistical inference, from the die rolls paradigm described above; but it is hard to see what the basis of this claim is.

Eppig et al. (2011) is one example of a study for which Pollet questions the use of *p*-values. In this paper, the researchers want to investigate the hypothesis that there is a developmental trade-off between maximizing brain vs. immune function. They argue that their theory predicts the existence of a correlation between average US state IQ and infectious disease stress. They observe the predicted positive correlation, using data from all 50 states and report the associated *p*-value. Contrary to what intuition might suggest, the population for which they are using inferential statistics is not the 50 US states. Eppig et al. are biologists, not US-states-ologists, so their object of study is presumably the human brain as shaped by evolutionary processes, not what happens to be the case in a certain country at an arbitrarily chosen point in time. The reason why they gather data on US states is because they aim at testing a general hypothesis about brains, and presumably find it convenient to use data that has been compiled for a country that merely happens to be the US. Considering the population for which the authors want to make inferences to be the 50 US states is similar to thinking that a psychology experiment done on undergraduates by a Yale psychologist has for population the set of psychology undergraduates at Yale.

Pollet argues that when using data from 50 US states to report a correlation at the level of US states, the situation is “unlike observations from rolling a dice, for example, where we can continue to roll a dice, and gather ever more observations” (p.1). It is unclear which argument actually lies beneath this claim. Clearly, “we can continue to roll a dice” cannot be interpreted in the sense of material possibility. If we run an experiment with a dice, but then destroy it, we are prevented from gathering further observations of this dice's behavior, but it would strike us as strange to conclude that the use of NHST is thereby forbidden. But if it cannot be interpreted in the sense of material possibility, then it is hard to see which kind of impossibility to gather new observations bars us from NHST.

To couch the point made by this commentary in more philosophical terms, when researchers are interested in more than mere description, their use of inferential statistics goes beyond addressing the problem of knowing whether they have sampled enough of the currently observable features of the world. They also have to face a mild version of Hume's problem of induction (Hume, 1748), that of using empirical data to draw inferences about causality. In this context, the definition of the studied population is less straightforward than what a literal interpretation of the term might suggest, and there is no reason to think that such a population is not very large compared to the sample.

I do not wish to defend the use of NHST in general, and happily acknowledge that its widespread use in the behavioral sciences can be damaging (Cohen, 1994; Loftus, 1996; Ziliak and McCloskey, 2008). Yet I do not think that issues of population finiteness should be a cause of specific concern for the relevance of NHST to cross-cultural research.

## Conflict of interest statement

The author declares that the research was conducted in the absence of any commercial or financial relationships that could be construed as a potential conflict of interest.
